# Importance of two-dimensional gaze analyses in the assessment of reading performance in patients with retinitis pigmentosa

**DOI:** 10.1371/journal.pone.0278682

**Published:** 2022-12-14

**Authors:** Masako Yoshida, Akitoshi Seiyama

**Affiliations:** 1 Yoshida Eye Clinic, Kyoto, Japan; 2 Division of Medical Devices for Diagnoses, Human Health Sciences, Graduate School of Medicine, Kyoto University, Kyoto, Japan; 3 Creative Design & Data Science Center, Akita International University, Akita-City, Akita, Japan; Justus Liebig Universitat Giessen, GERMANY

## Abstract

The causes of reading difficulties in people with peripheral visual field loss are not fully understood. We conducted a two-dimensional gaze analysis on eye movements during reading in patients with retinitis pigmentosa to investigate the causes of reading difficulties in relation to the central visual field using a binocular eye mark recorder (EMR-9). Twenty-seven patients with retinitis pigmentosa whose central visual field narrowed to ≤ 20° using Goldmann kinetic perimetry (I/4 target) and this present study included eight healthy participants. The participants’ visual acuities were corrected to better than +0.4 logMAR. Correlations and multivariate regression analyses were investigated between the number of letters read correctly, the I/4 central visual field, V/4 perifoveal and peripheral visual field, and visual acuity. Multivariate regression analysis revealed that all these parameters played almost equal roles in the number of letters read correctly. In the two-dimensional gaze analysis, the task performance time of patients during reading increased as the I/4 central visual field narrowed. The task performance time was more clearly correlated with the rotation saccade (r = 0.428, p <0.05) and the distance of the vertical direction (ΣY) of eye movements (r = 0.624, p < 0.01), but not with regressive saccade and the distance of the horizontal direction (ΣX). Visual acuity was correlated with the task performance time (-0.436, <0.05) but not with eye movement directionality. Reading difficulties in patients with retinitis pigmentosa result from impaired eye movement directionality. Understanding eye measurements for people with tunnel vision required a two-dimensional gaze analysis. The two-dimensional gaze analysis also showed that the involvement of the perifoveal and peripheral visual fields, visual acuity, and I/4 central visual field was important for reading in people with tunnel vision.

## Introduction

Retinitis pigmentosa (RP) is one of the major diseases associated with peripheral visual field loss; it is a hereditary progressive disease that occurs in approximately 1 in 4,000 people. In many cases, the disease initially affects the rod cells near the macula; therefore, visual field impairment in patients with RP involves isolated scotomas near the macula, expanding and fusing to form a ring-shaped scotoma as the disease progresses. Further progression results in peripheral visual field loss due to the enlargement of the ring-shaped scotoma, leading to the concentric narrowing of the visual field [1]. Therefore, patients with RP struggle in daily life to determine the entire visual field due to the narrowing of their central visual field and have difficulty searching for things and moving due to the loss of the peripheral visual field [1–3]. If the central visual field narrows to a diameter of ≤ 20°, reading may become difficult due to character oversight or line breaks, even though the individual’s eyesight is sufficient for reading characters [4, 5].

A countermeasure to reading difficulties includes improving the visibility of characters, such as using a magnifying glass to deal with the deterioration of visual acuity. However, even if visual acuity is normal, narrowing of the central visual field results in reading difficulties because of line breaks resulting in errors or difficulty tracking characters. Furthermore, no effective countermeasures have been established for reading difficulties in people with tunnel vision other than indirect methods such as visual positioning with underlines or a typoscope.

This research area has been the subject of many studies ever since Javal first proposed in 1879 that eye movements in reading were repetitions of fixation and saccade (SC) [6]. There have been reports of gaze measurements of glaucoma patients who first experience a disappearance of hemi-peripheral vision and eventually have impaired foveal vision [7–12]. All these previous studies of eye movements during reading were analyses of latency, amplitude, forward and regressive frequencies, retention frequency, and mean duration, but only for horizontal SCs with adjustments for calibration errors. However, there are no reports of detailed gaze measurements in reading for patients with RP.

We previously used functional magnetic resonance imaging to investigate the eye movement-related brain regions in patients with advanced RP [13]. The impaired SCs in patients with advanced RP were caused by decreased activity in eye movement-related brain regions in the frontal and parietal eye fields due to decreased visual input from the retina. An SC is related to controlling the direction and distance of the line-of-sight movement from the peripheral visual field to the fovea centralis. Therefore, there is a need to analyze the movement direction and distance of the line of sight for eye movements during reading in patients with advanced RP accompanied by peripheral visual field loss and central visual field narrowing.

Patients with advanced RP who visit the first author’s Yoshida Eye Clinic often state that they skip the next line or lose track of the beginning of the next line in sentence line breaks. They also state that unintended movements of the viewpoint to another line, even in the middle of a line, cause the individual to lose sight of the part where the next viewpoint is placed, and their reading is interrupted.

Based on the results of functional magnetic resonance imaging studies and the reports of patients with advanced RP, we hypothesized that "directionalization of SCs" would cause reading difficulties in patients with advanced RP who otherwise had normal visual acuity. This study aimed to elucidate the cause of reading difficulties in patients with advanced RP using two-dimensional gaze analysis.

## Materials and methods

### Participants

This study was approved by the Ethics Committee of Kyoto University Graduate School and Faculty of Medicine, and both written and verbal informed consents were obtained from the participants. Participants were adults up to 65 years of age with sufficient vocabulary and language skills with at least 12 or more years of education. They included patients with RP and a central visual field that was narrowed to ≤ 20° using Goldmann kinetic perimetry (GKP) (I/4 target) and healthy participants. The criteria for inclusion in the study were that the participants had to bitually read books and had no problems conducting this task. During this study, at least one eye was corrected to +0.4 LogMAR or better as a level of visual acuity that could identify characters in reading material. The reliability of the results of gaze analysis was ensured by excluding patients who could not satisfy the calibration criteria of the measuring instrument. In total, 27 individuals (12 men, 15 women) aged 22–64 years (average 42.2 ± 12.7 years) satisfied these conditions. The control group comprised eight healthy adults of a similar age to the patients with RP and had no ophthalmologic abnormalities (four men and four women, aged 25–62 years, average 44.5 ± 13.7 years; see S1 Table).

### Analysis of visual function tests

In the case of non-synkinetic movements such as congestion and diversion, the left and right eyes do not always move in a synkinetic manner [14, 15]. The eye movements were assessed simultaneously with both eyes open. The main fixating eye was identified as the eye with a high rate of matching with the line of text. We investigated the visual field (Goldmann kinetic perimetry, GKP), visual acuity, and the number of character reads in 35 fixating eyes in 27 patients with advanced RP and eight healthy participants.

### Goldmann kinetic perimetry

The visual function of the participants was evaluated based on the central visual field of the I/4 target of the GKP. The size of the central visual field of the GKP (I/4 target) in the participants was used to divide them into the following four groups: central visual field < 5° in one eye (advanced), ≥ 5° and < 10° (moderate), ≥ 10° and ≤ 20° (mild), and healthy controls.

It has been reported that peripheral vision affects the fixation/SC pattern during reading [16]. Therefore, we also conducted evaluations of the peripheral visual field using GKP (V/4 target). The areas covered by a radius of 5–10° and > 10° with a V/4 target were set as the perifovea and periphery, respectively; the area retention rates of the perifoveal and peripheral visual fields of the participant were calculated for each area, with the area retained by healthy individuals as 100%.

### Visual acuity test (5-m distance visual acuity and near visual acuity)

We measured LogMAR visual acuity using a 5-m distance vision chart (SC-2000, 4987669605011, NIDEK, Tokyo, Japan) and a 30-cm near vision chart (Yamachi Chart for Near Point Distance, HANDAYA, Tokyo, Japan).

### Reading speed

We investigated reading speed under free reading conditions. The number of characters read aloud in 5 min was used as an index of reading ability. The reading material had 22 characters per line and 18 lines per page on white A4 paper, and a plain horizontal Japanese text using both kanji and kana was used. The characters used were in Mincho 20-point font (viewing angle 0.857°, composition line 0.037°), which is commonly used in a book. Participants were asked to read aloud with both eyes open at a viewing distance of 40 cm. The experimenter counted the number of characters that could be accurately read for 5 min. It was conducted three times from different parts of the book, and the average value was adopted as the number of characters read.

### Analysis of ocular motility

#### Task and eye movement recording

The line-of-sight measurements were performed in an indoor environment adjusted to an illuminance of 1000 Lx with a fluorescent lamp. The task used a Japanese text of 35 characters per line and 15 lines in a horizontal direction per page, which consisted of both kanji and kana in a 20-point MS Gothic typeface to avoid the effects of low visual acuity on character readability. Participants were instructed to read aloud the horizontal lines of text displayed on the monitor screen at a speed at which they could achieve the task without a time limit.

A head-fixing device with a chin rest was used. The device was placed so that the participants’ eye height aligned with the monitor’s center. Participants could only move their eyes while reading. The distance between the apex of the cornea and the center of the screen during the test was maintained at 40 cm.

A binocular eye mark recorder (EMR-9 Eye Tracking System; NAC Image Technology, Minato, Tokyo, Japan) was used for the recordings. The sampling rate of the eye-tracking system was 0.1° (60 Hz). The calibration function of the EMR-9 eye-tracking system was used to identify nine gaze points on the monitor screen with a viewing angle of 37°. Calibration was repeated until the instrument calibration criteria were met.

#### Analyses

The obtained data (see S2 Table) were analyzed using EMR-d Factory, which is included in the EMR-9 eye-tracking system. One line at eye level was analyzed.

#### Trajectory of fixation

**Fig 1:**
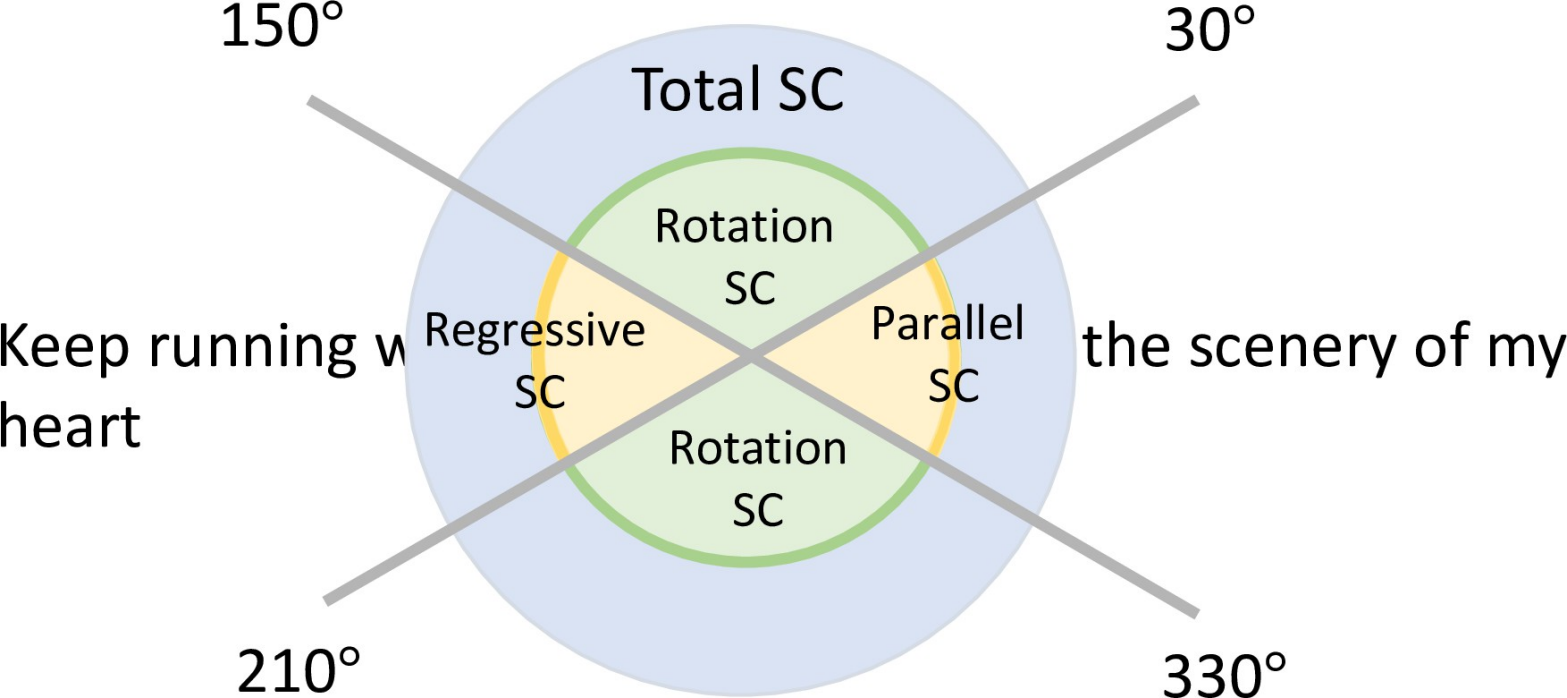
Definition of visual directions in the present study. Abbreviations: SC, saccade. The angle between 30° and 330°and that between 150° and 210° were defined as the parallel SC. The angles between 30° and 150° and that between 210° and 330° were defined as rotation SC.

Fixation was defined as the stationary gaze point for 0.1 s or more. The movement angle between fixation was automatically classified and recorded. Any movement in which the movement angle between fixation was within 30° above or below the line of characters was defined as parallel SCs (see Fig 1). Parallel SCs in the progressing direction of the line of characters was defined as progression, and opposite to the progressing direction of the line of characters was defined as regression. A movement angle ≥ 30° from the line of text was defined as the rotation SC. We identified the position of the gaze point by voice information, and the eyes with a higher rate of matching the gaze point with the line of text were judged as the dominant eye.

The analysis items (Fig 1) were the regressive SC, the rotation SC with the line of text as the baseline (0°) for rotational movement, total eye movement distance by direction (ΣX, ΣY), and task performance time. The proportions of the regressive SC and rotation SC were calculated with all SCs (including SCs that were parallel to the line of text and SCs with movement in the rotation SC) set as 100%. The correlations between the analysis results for each item and the I/4 central visual field, the V/4 visual field retention rate, visual acuity, and age were investigated.

We evaluated the number of fixations for a given analyzed line (no. of fixation), mean duration of fixation per line (mean duration of fixation), the proportion of regression in all movements between fixation per line (regressive SC), and the proportion of movements in the rotation direction (rotation SC).

### Trajectory of eye movement

A quantitative analysis of the trajectory of eye movement was conducted. Here, eye movements in various directions between each fixation were recorded for all the participants. Therefore, instead of the shortest distance between fixation, the eye movement trajectory was divided into units of 0.01 s to precisely measure the total movement distance.

The distance of eye movement (unit viewing angle) was measured separately for the horizontal component (X) and the vertical component (Y). The total movement distance by direction (ΣX = Σ(*x*_*i*_−*x*_*i*−1_), ΣY = Σ(*y*_*i*_−*y*_*i*−1_), and the sum of the total distances ΣTotal (= $\Sigma \sqrt{{\left(x_{i}-x_{i-1}\right)}^{2}+{\left(y_{i}-y_{i-1}\right)}^{2}}$ were calculated.

Cases where the gaze point was not on the screen resulted in the X and Y directions being recognized as out of the measurement range. The data were automatically listed as "error" and "unable to measure eye movement" and excluded from the analysis. The error frequency was used as a reference item because it caused an underestimation of the eye movement distance.

### Task performance time (s)

The time (s) required for eye movement across one line, analyzed at eye height, was recorded to evaluate the relationship between eye movement and reading speed. Cases, where lines were skipped were excluded from the analysis target, and measurements were conducted on lines that were read without skipping.

### Statistical analyses

The participant’s visual field and gaze analysis data are shown in Tables 1 and 2, respectively, using means and standard deviations. The following statistical analyses were performed using statistical software (College Analysis ver. 8.5) [17]. The Kruskal Wallis H test and the Wilcoxon joint test were used for comparative tests between each group, and the significance level was set at 5%. The partial least squares regression was used to contribute to the individual visual field function on the number of letters read correctly. Pearson correlation analysis and linear regression were used for the correlation analysis between data. The strength of the correlation was defined as follows: a correlation coefficient (± r) ≥ 0.7 was set as a strong correlation, 0.4–0.69 as a moderate correlation, 0.2–0.39 as a weak correlation, and < 0.2 as no correlation.

**Table 1:** Visual function tests in 35 eyes of 35 participants.

	advanced	moderate	mild	Healthy
	n = 9	n = 9	n = 9	n = 8
**I/4 central**	3.61 ± 0.84	6.67 ± 0.90	14.56 ± 1.86*	100 ± 0*^,+^
**(degree)**				
**V/4 perifovea**	28.44 ± 6.00	47.78 ± 6.81	85.67 ± 5.05*^,+^	100 ± 0*^,+^
**(%)**				
**V/4 periphery**	10.11 ± 3.67^#^	14.22 ± 4.25^#^	25.22 ± 4.96^#^	100 ± 0
**(%)**				
**Acuity**	0.31 ± 0.33	0.19 ± 0.41	0.12 ± 0.35*^,^^#^	‵-0.16 ± 0.26*^,+^
**(LogMAR)**				
**Age**	46.89 ± 3.15	42.89 ± 3.79	39.89 ± 3.51	44.50 ± 3.70
**(year)**				
**Number of letters correctly read**	1,026 ± 18	1,623 ± 25	1,780 ± 15*	2,019 ± 16*

The Kruskal Wallis H test and the Wilcoxon joint test were used.

*, p < 0.05 vs. advanced, +, p < 0.05 vs. moderate

#, p < 0.05 vs. healthy

**Table 2:** Results of the eye movement test using EMR-9.

	advanced	Moderate	mild	healthy
	n = 9	n = 9	n = 9	n = 8
**Number of fixations**	15.33 ± 1.59	10.67 ± 0.59*	9.67 ± 0.27**	10.0 ± 0.50*
**Mean duration of fixations**	0.60 ± 0.11	0.51 ± 0.04	0.58 ± 0.04	0.57 ± 0.03
**Regressive SC (%)**	18.87 ± 3.07	21.83 ± 3.43	18.76 ± 2.67	17.33 ± 2.35
**Rotation SC (%)**	29.54 ± 9.86	16.51 ± 6.69	11.3 ± 5.13	4.0 ± 2.45*
**ΣtTotal (degree)**	236.02 ± 11.06	118.15 ± 4.38**	134.05 ± 6.42*	109.88 ± 5.08**
**ΣX (degree)**	201.86 ± 11.56	100.52 ± 5.75*	111.34±7.39	81.41 ± 3.38**
**ΣY (degree)**	170.86 ± 8.63	64.73 ± 4.48	58.73 ± 5.33*	48.48 ± 4.88**
**Task performance time (s)**	10.13 ± 1.96	6.40 ± 0.97**	6.12 ± 0.92**	6.70 ± 0.81*

ΣX (degree); total eye movement distance in the horizontal direction; ΣY (degree); total eye movement distance in the vertical direction.

The Kruskal Wallis H test and the Wilcoxon joint test were used.

*, p < 0.05 vs. advanced group

**, p < 0.01 vs. advanced group

## Results

### Analysis of visual function tests

Table 1 shows visual function according to the central visual field group by GKP (I/4 target) for the fixating eye of the subject. The mean I/4 central visual field for the group was as follows: I/4 central visual field < 5° (advanced), 3.6° ± 0.84 S.E.; ≥ 5° and < 10° (moderate), 6.7° ± 0.90 S.E.; ≥ 10° and ≤ 20° (mild), 14.6° ± 9 S.E.; and healthy group (healthy), 100° ± 0 S.E. There were significant differences between the advanced group and the mild and healthy groups.

The number of characters read aloud for 5 minutes was also significantly smaller at 1,026 characters (± 18 S.E.) for the advanced group than that of the healthy group at 1,980 characters (± 95 S.E.).

The multivariate regression analysis (partial least squares regression) applied to Table 1 (but without age and healthy control) gave the following regression formula with standardized partial regression coefficients:

Numberoflettersreadcorrectly=0.217*(I/4central)+0.226*(V/4perifovea)+0.216*(V/4periphery)–0.255*(acuity)

The coefficients of determination and residual variance were 0.811 and 19925, respectively.

Further, regression analyses of these parameters and the number of letters read correctly for 27 patients suggested that the V/4 perifoveal (r = 0.384, p < 0.05) and V/4 peripheral (r = 0.435, p < 0.05) visual fields, as well as the visual acuity (r = –0.542, p < 0.01) and I/4 central visual field (r = 0.514, p < 0.01), were involved in the number of characters read (cf., S2 Fig).

### Gaze analysis

During reading in healthy participants, the eye movements were parallel to the line of characters, whereas the eye movement in the patients with RP was often at irregular angles with the line of characters. As the I/4 central visual field narrowed further or the visual field disappeared further, the frequency of error judgments in which the gaze point popped out of the recording screen increased.

**Fig 2:**
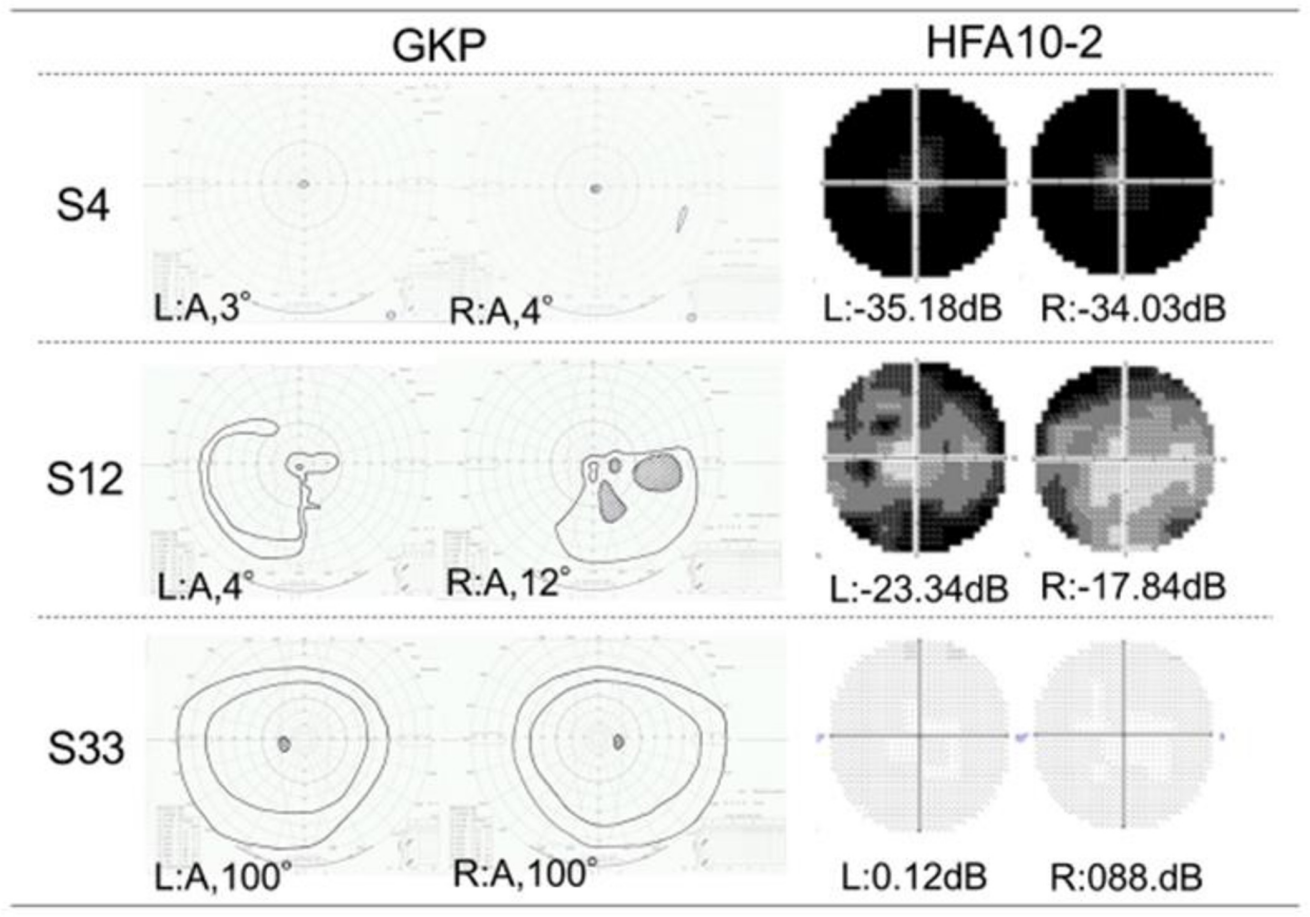
Typical examples of VFs measured using GKP and HFA 10–2. VFs measured using GKP V/4 & I/4 (right) and HFA 10–2 (left). The eye classification and pathology of each subject (S4, S12, and S33) are shown in the S1 Table. Abbreviations: GKP, Goldmann kinetic perimetry; HFA 10–2, Humphrey static perimetry.

**Fig 3:**
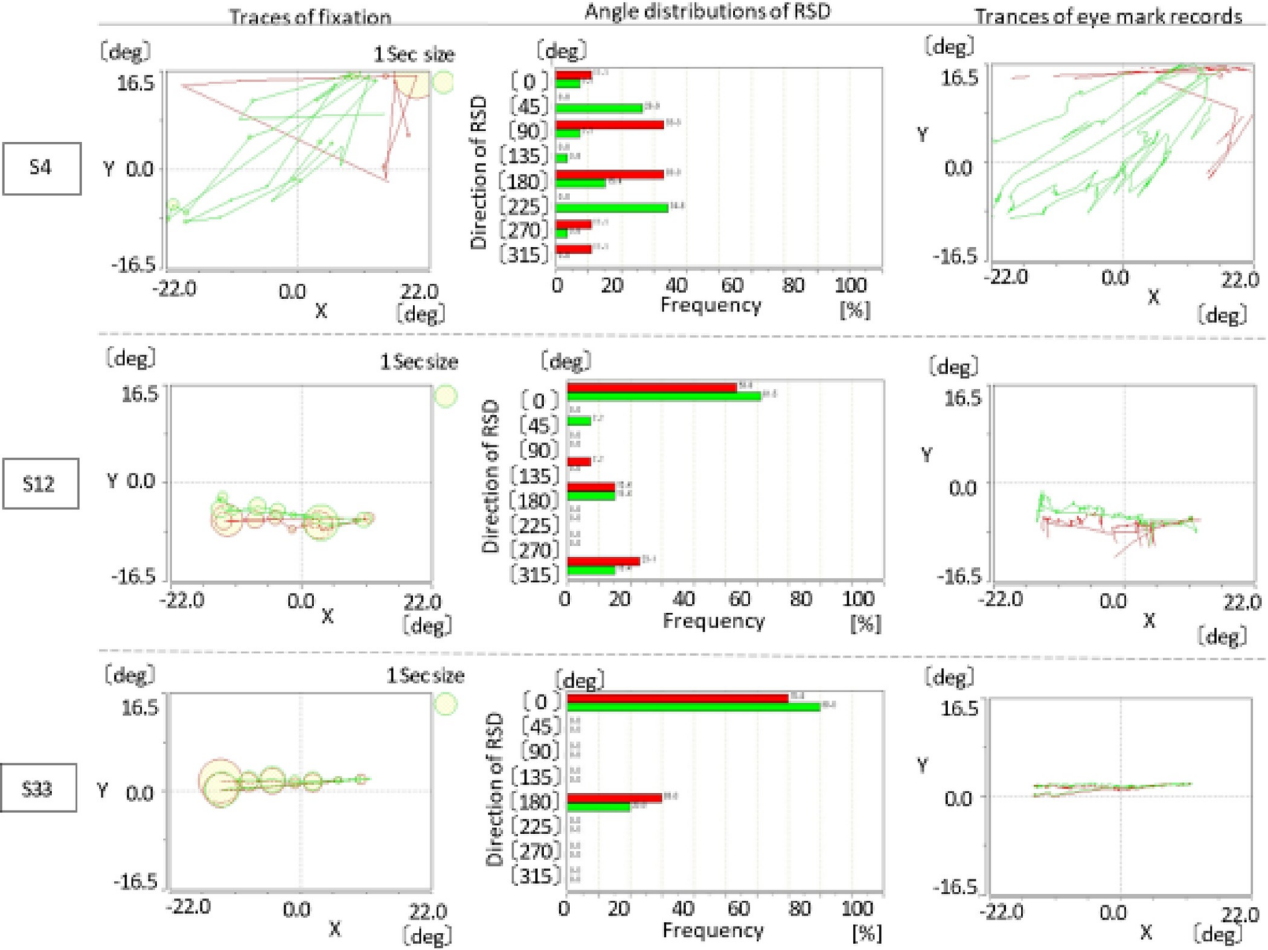
Typical examples of analyses of eye movements during reading. S4 traces for a patient with a visual field of 4° in the right eye (the green line) and 3° in the left eye (the red line). S12 traces for a patient with a visual field of 10° in the right eye (the green line) and 4° in the left eye (the red line). S33 traces for a healthy volunteer with a visual field of 100° in both eyes (right: the green line, left: the red line. **Left**: Traces of fixation. The size of the circle indicates the length of the residence time at that position. The red lines and circles reflect the movement of the left eye, and the green lines and circles reflect the movement of the right eye. **Middle**: Angle distribution of the rotation SC direction. The red and green bars denote the left and right eyes, respectively. **Right**: Traces of eye movements every 0.01 s. The red and green lines denote the traces of the left and right eyes, respectively. Abbreviations: SC, saccade.

For the frequency of movement between fixation, rotation SC increased with the narrowing of the I/4 central visual field. Fig 2 shows the retained visual fields of individuals with highly advanced RP (S4), moderately advanced RP (S12), and healthy individuals (S33); Fig 3 shows the images for the trajectory of fixation as well as the frequency by direction of movement between fixation and the trajectory of eye movement.

### Trajectory of eye movement and task performance time

Table 2 summarizes the analysis results of the trajectory of eye movement. The proportion of rotation SC during the fixation trajectory increased inversely to the narrowing of the I/4 central visual field, with a significant (p < 0.05) difference between the advanced group (29.54% ± 9.86) and the healthy group (4.0% ± 2.45). Meanwhile, there were no significant differences between groups in the mean duration of fixation and regressive SC.

The eye movement distance was underestimated due to the increased frequency of error (gaze points outside the screen) owing to a narrowed I/4 central visual field. However, there were still significant (p < 0.05) differences in all eye movement distances (ΣX, ΣY, ΣTotal) between the advanced and the healthy groups. The task performance time increased with the narrowing of the I/4 central visual field, and there were significant (p < 0.05) differences between the healthy group (6.70 s ± 0.81 S.E.) and the advanced group (10.13 s ± 1.96 S.E.).

### Individual gaze analysis and visual function of RP patients

Table 3 shows the correlations between the gaze analysis results (trajectory of fixation and trajectory of eye movement) in 27 fixating eyes with a narrowed I/4 central visual field and the task performance time, visual function (I/4 central visual field, V/4 perifoveal visual field, V/4 peripheral visual field, and visual acuity).

**Table 3:** Correlation coefficients between parameters of visual function and eye movement analysis.

I/4 central visual field below 20		Analysis of ocular motility							
		Trajectory of fixation				Trajectory of eye movement			Task performance time
		No of FX	FX duration	Regressive SC	Rotation SC	ΣTotal	ΣX	ΣY	
Task performance time		0.385*	0.418*	-0.303	0.428*	0.385*	0.293	0.624**	-
Visual field	I/4 central	-0.459*	-0.026	0.022	0.317	-0.401*	-0.357	-0.448*	-0.486*
	V/4 perifovea	-0.386*	-0.169	0.189	-0.42	-0.397*	-0.373	-0.416*	-0.492**
	V/4 periphery	-0.316	(-)	0.194	-0.234	-0.043	-0.004	-0.248	-0.270
Acuity		0.207	0.125	0.102	-0.003	0.272	0.205	0.199	0.436*
Age		0.116	0.079	0.022	0.212	0.27	0.155	-0.041	0.058

1/4 central, 1/4 central visual field; V/4 perifovea, V/4 perifovea visual field; V/4 periphery, V/4 periphery visual field; Acuity, LogMAR; No of FX, number of fixations; FX duration, mean duration of fixation. We defined the significance levels of the correlation coefficients as follows: a correlation coefficient of ≥ 0.7 was defined as a " strong correlation," that of 0.4–0.69 as a "moderate correlation," that of 0.2–0.39 as a " weak correlation," and that of < 0.2 as "no correlation."

*, p < 0.05 vs. advanced group

**, p < 0.01 vs. advanced group.

### Gaze analysis and visual field

The correlation between visual function and gaze analysis results was evident in the vertical direction of eye movements. In the trajectory of fixation, all visual fields were negatively correlated with the rotation SC. On the contrary, there were no correlations between any of the visual fields with parallel regressive SC.

In addition, for the trajectory of eye movement, there was a significant moderate correlation between the I/4 central and V/4 perifoveal visual fields and the eye movement distance in the vertical direction ΣY, but not with the eye movement distance in the horizontal direction ΣX.

All visual fields were significantly negatively correlated with the number of fixations, but there were no correlations between any visual fields and the mean fixation duration.

### Visual acuity and gaze analysis

Visual acuity was moderately significantly correlated with total performance time only, while age showed no significant correlation with the ocular motility parameters. The eye movement abnormalities, however, exhibited by the patients in this study did not depend on the age at onset and the duration of onset.

## Discussion

In this study, two-dimensional gaze analysis showed, for the first time, that the vertical component that deviated from the line of characters increased with the narrowing of the I/4 central visual field in eye movement during reading in patients with RP.

Reading is a repetition of fixation and SC. Saccades are triggered by position information of the peripheral retina; thus, the loss of the peripheral visual field is not limited to the lack of position information on the peripheral retina, which leads to impairment of the control system for eye movement direction and distance. Makiyama et al. reported that rod and pyramidal cells were damaged in the early stage of RP [18]. Loss of cells in the perifoveal region leads to increased modified SCs, likely increasing eye movement distance. Therefore, gaze analysis during reading in patients with RP requires the analysis of the movement angle between fixation as a means for expressing the deviation from lines of characters and the total eye movement distance as a means for expressing character oversights.

In this study, narrowed I/4 central visual field of patients with RP showed relatively higher correlations between all visual fields and the rotation SC of the trajectory of fixation than those between all visual fields and the regressive SC. Furthermore, there was a relatively stronger correlation between all visual fields and the vertical component (ΣY) rather than the horizontal component (ΣX) of the total eye movement distance. These results suggest that abnormalities in the vertical direction may be more prominent in patients with RP and that two-dimensional gaze analysis is necessary for patients with RP.

Additionally, in the one-line fixation trajectory of the gaze task, task performance time had a significant moderate correlation with rotation SC but not with regressive SC. Furthermore, the eye movement trajectory correlated significantly with task performance time for the total eye movement distance in the vertical direction ΣY but not for the total eye movement distance in the horizontal direction ΣX. The data indicate that the decrease in reading speed in patients with advanced RP is partly due to the increase in the vertical component in eye movement.

These results from a two-dimensional analysis of eye movements during reading support our hypothesis that eye movement in rotation directions causes reading difficulties in patients with advanced RP.

All previous studies of eye movements during reading were conducted on glaucoma patients with one-dimensional gaze analyses [7–12]. This time, the increase in movement in rotation directions (i.e., rotation SC) in patients with RP showed the strongest correlation with the V/4 perifoveal visual field, followed by the I/4 central visual field and V/4 peripheral visual field (Table 3). This suggests that even patients with glaucoma, whose disease starts from the peripheral visual field and results in the disappearance of the perifoveal visual field, may exhibit eye movement in rotation directions during reading, similar to patients with RP. However, glaucoma patients who retain peripheral vision may have a lower frequency of eye movements in rotation directions than patients with RP.

Reading involves foveal vision and peripheral vision [16, 19]. Rayner [19] and Ikeda et al. [4] reported that visual information processing in the parafoveal area with a viewing angle of 8° is important for determining the landing position of the next gaze point. The results in the present study, where, as with the I/4 central visual field, the V/4 perifoveal visual field was correlated with the extension of the total eye movement distance, suggest the relationship between the foveal vision and peripheral vision.

Ikeda et al. reported that the visual information of the parafoveal area affected the fixation duration [4]. However, the present study did not show any correlation between the visual field and fixation duration. However, a report indicates that although not accompanied by a decrease in reading speed, the mean duration of fixation, which was correlated with the static visual field indicator HFA10-2, was extended in glaucoma patients [12]. Differences from previous research results may be due to differences in the visual field assessments used. In the future, there is a need to proceed with eye movement analyses on RP cases in the I/4 central visual field < 5° group and investigate the correlations between the HFA10-2-based retinal sensitivity distribution and GPK-based viewing angle (I/4 central visual field and V/4 perifoveal visual field) and eye movement.

The number of fixations gave a significant and strongest correlation with the I/4 central visual field and showed a significant but weak correlation with the task performance time. The increase in the number of fixations during reading in tunnel vision is consistent with the findings of the reports by Smith et al. [8, 9] and Murata et al. [12].

According to Morrison and Rayner [20], SC size was determined not by the viewing angle but by the letter spacing. Regarding the number of characters that can be seen and reading speed, Legge et al. [21] reported a decrease in reading speed with four characters or less. Osaka and Oda [22] reported a decrease in reading speed with five characters or less in Japanese with a mixture of kanji and kana characters. The printed reading material (viewing angle 0.857°) and gaze analysis task (viewing angle 1.045°) used in the present study had five consecutive characters with a viewing angle of around 5°. The results in the present study, where the decrease in the number of characters read and extension of task performance time according to the I/4 central visual field was only significant (p < 0.05) in the I/4 central visual field of < 5° group (advanced), were consistent with the results in the above-mentioned report.

In addition to the foveal viewing angle, several factors, such as visual acuity, contrast sensitivity [9, 23], and working memory [24], have been associated with reading ability. Research using static perimeters by Virgili et al. [5] showed that reading speed in patients with RP was correlated with visual acuity and contrast sensitivity and that the correlation was strongest with a mean retinal sensitivity within 6° of the fovea. Sandberg et al. [25] reported that the reading speed in patients with RP was correlated only with contrast sensitivity, not with the foveal visual field diameter. The gaze analysis task involved a display of easy-to-read Gothic font in 100% contrast to control for the effects of low vision and reduced retinal sensitivity. Multivariant regression and correlation analyses revealed that the visual acuity and I/4 central visual field played major roles in the number of letters read correctly. Further, the involvement of the perifoveal visual field and peripheral visual field were shown during reading of RP patients.

Our study had several limitations. The patients enrolled in this study were relatively young, so our findings cannot be generalized to older patients; thus, further studies that include older individuals are necessary. In addition, the number of patients in the advanced group was small; larger sample sizes are needed to identify significant correlations.

## Conclusion

The present study clarified for the first time that reading difficulties in patients with RP are due to SC disorders in mainly vertical rotation SC. This result supports the notion that two-dimensional analysis is required for gaze measurements during reading in patients with RP. Our results also suggest that the involvement of the perifoveal visual field and peripheral visual field, as well as visual acuity and I/4 central visual field, is important for eye movement during reading. These are important findings that could elucidate the need for ophthalmic rehabilitation for patients with RP.

## Supporting information

S1 Fig Typical example of correlation analyses between gaze analyses and visual field. (A) Percentage (%) of the regression saccade (SC) relative to the total SC direction. (B) Percentage (%) of the rotation saccade>30° (RSD>30) relative to the total SC direction. (C) Number of fixations. (D) Duration(s) of fixation (> 0.1 s). The data of the 27 patients are presented.
(PPTX)

S2 Fig Correlation analyses between number of letters correctly read and visual function in 27 patients. The graphs show the relationship between number of letters correctly read vs the I/4 central visual field (A), vs V/4 perifovea (B), vs V/4 periphery (C) and vs acuity (D).
(PPTX)

S1 Table Analysis of ocular motility in our study population. (DOCX)

S2 Table Visual function measured with an eye mark recorder for all participants. (DOCX)
