# Supplementary material for: Importance of two-dimensional gaze analyses in the assessment of reading performance in patients with retinitis pigmentosa
Source: PLoS One. 2022 Dec 14;17(12):e0278682. doi: 10.1371/journal.pone.0278682 (PMC9750004; doi:10.1371/journal.pone.0278682)
Supplement: S1 Fig — (A) Percentage (%) of the regression saccade (SC) relative to the total SC direction. (B) Percentage (%) of the rotation saccade>30° (RSD>30) relative to the total SC direction. (C) Number of fixations. (D) Duration(s) of fixation (> 0.1 s). The data of the 27 patients are presented. (PPTX) [file pone.0278682.s001.pptx]

## Slide 1
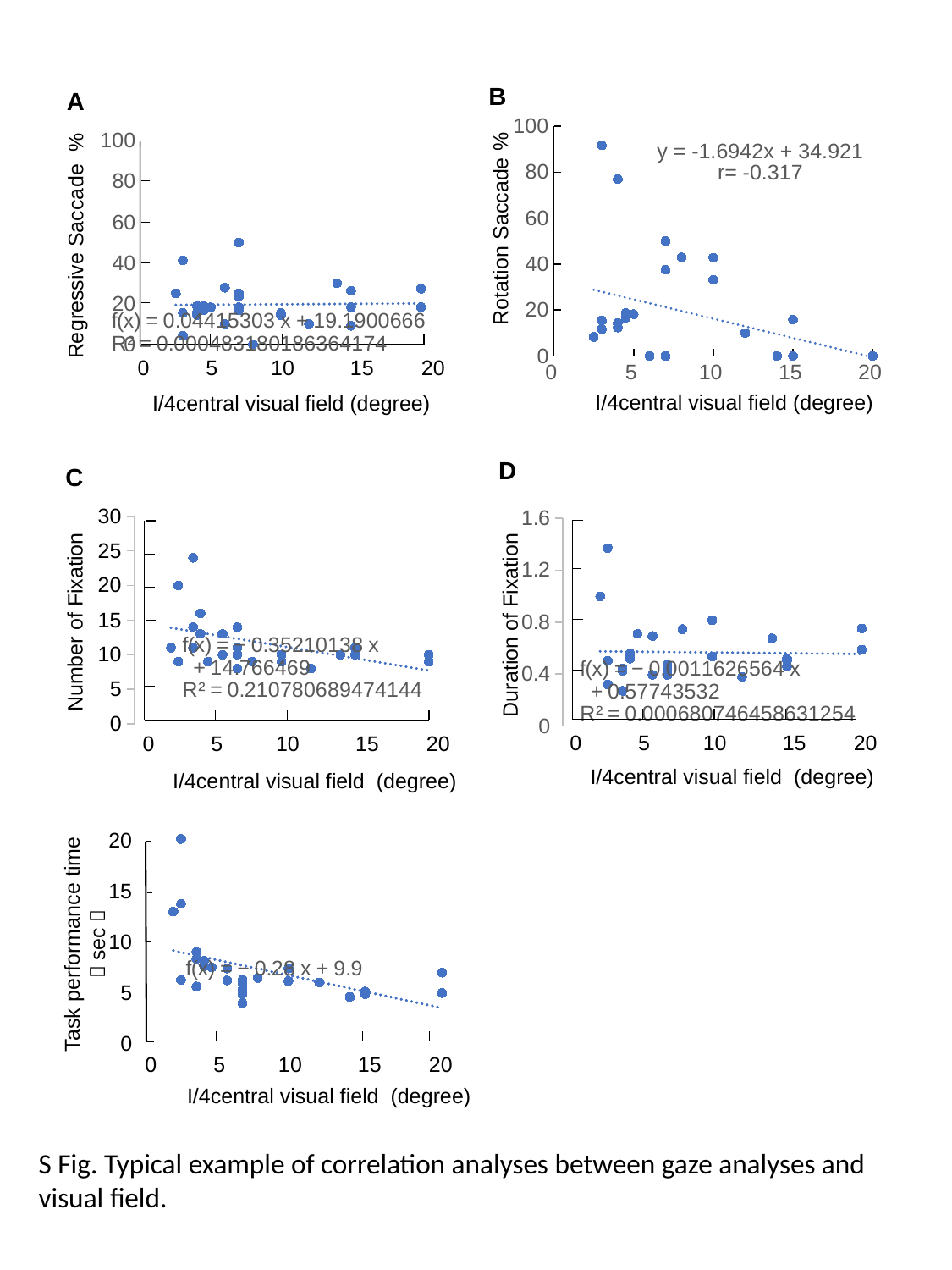

B
### Chart
| Category | 他方向 |
|---|---|Rotation Saccade %
y = -1.6942x + 34.921
r= -0.317
I/4central visual field (degree)
A
### Chart
| Category | |
|---|---|0 5 10 15 20
I/4central visual field (degree)
 Regressive Saccade %
D
### Chart
| Category | |
|---|---|0 5 10 15 20
I/4central visual field (degree)
 Duration of Fixation
C
### Chart
| Category | |
|---|---|0 5 10 15 20
I/4central visual field (degree)
 Number of Fixation
20
15
10
5
0
### Chart
| Category | |
|---|---|0 5 10 15 20
Task performance time
（sec）
I/4central visual field (degree)
S Fig. Typical example of correlation analyses between gaze analyses and visual field.
