# Supplementary material for: Importance of two-dimensional gaze analyses in the assessment of reading performance in patients with retinitis pigmentosa
Source: PLoS One. 2022 Dec 14;17(12):e0278682. doi: 10.1371/journal.pone.0278682 (PMC9750004; doi:10.1371/journal.pone.0278682)
Supplement: S2 Fig — The graphs show the relationship between number of letters correctly read vs the I/4 central visual field (A), vs V/4 perifovea (B), vs V/4 periphery (C) and vs acuity (D). (PPTX) [file pone.0278682.s002.pptx]

## Slide 1
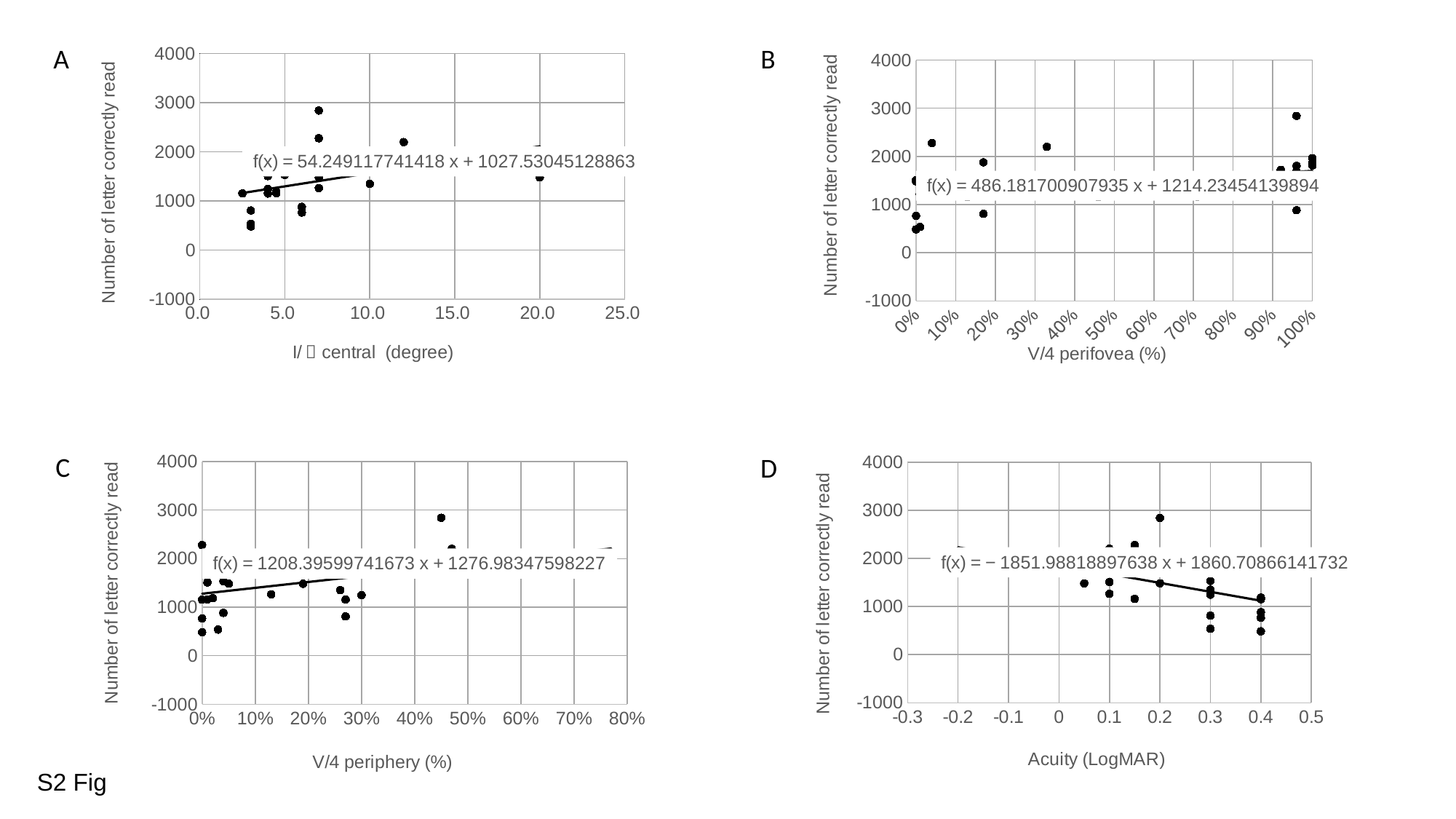

A
### Chart
| Category | Number of letter correctly read | | | | |
|---|---|---|---|---|---|B
### Chart
| Category | Number of letter correctly read | | | | |
|---|---|---|---|---|---|C
### Chart
| Category | Number of letter correctly read | | | | |
|---|---|---|---|---|---|
### Chart
| Category | Number of letter correctly read | | | | |
|---|---|---|---|---|---|D
S2 Fig
