# Supplementary material for: Importance of two-dimensional gaze analyses in the assessment of reading performance in patients with retinitis pigmentosa
Source: PLoS One. 2022 Dec 14;17(12):e0278682. doi: 10.1371/journal.pone.0278682 (PMC9750004; doi:10.1371/journal.pone.0278682)
Supplement: S1 Table — (DOCX) [file pone.0278682.s003.docx]

**S1 Table. Visual function in our study population.**

| Subject No. | Gender | Age (years) | Number of letter correctly read | Eye | Group | Visual field | | | | LogMAR | |
| --- | --- | --- | --- | --- | --- | --- | --- | --- | --- | --- | --- |
|  |  |  |  |  |  | I/4 central viewing angle (degrees) | V/4 | | |  |  |
|  |  |  |  |  |  |  | Perifovea  (%) | Periphery  (%) |  | |  |
|  |  |  |  |  |  |  |  |  |  |  |  |
| S1 | F | 43 | 537 | R | ADVANCED | 3.0 | 1% | 3% | 0.3 | |  |
|  |  |  |  | L | ADVANCED | 1.0 | 0% | 4% | 0.5 | |  |
| S2 | M | 38 | 484 | R | ADVANCED | 3.0 | 0% | 0% | 0.4 | |  |
|  |  |  |  | L | ADVANCED | 1.5 | 0% | 0% | 0.4 | |  |
| S3 | M | 41 | 1155 | R | ADVANCED | 2.5 | 13% | 27% | 0.4 | |  |
|  |  |  |  | L | ADVANCED | 2.5 | 13% | 38% | 0.5 | |  |
| S4 | F | 37 | 1509 | R | ADVANCED | 4.0 | 0% | 1% | 0.1 | |  |
|  |  |  |  | L | ADVANCED | 3.0 | 0% | 0% | 0.1 | |  |
| S5 | M | 63 | 1154 | R | ADVANCED | 3.0 | 42% | 0% | 0.5 | |  |
|  |  |  |  | L | ADVANCED | 4.0 | 46% | 0% | 0.4 | |  |
| S6 | F | 55 | 1160 | R | ADVANCED | 3.0 | 50% | 0% | 0.2 | |  |
|  |  |  |  | L | ADVANCED | 4.5 | 71% | 1% | 0.15 | |  |
| S7 | F | 63 | 808 | R | ADVANCED | 3.0 | 17% | 27% | 0.3 | |  |
|  |  |  |  | L | MODERATE | 5.0 | 21% | 35% | 0.3 | |  |
| S8 | M | 64 | 1483 | R | ADVANCED | 3.0 | 0% | 1% | 0.4 | |  |
|  |  |  |  | L | MODERATE | 7.0 | 0% | 5% | 0.2 | |  |
| S9 | F | 41 | 1246 | R | ADVANCED | 4.0 | 100% | 30% | 0.3 | |  |
|  |  |  |  | L | ADVANCED | 4.0 | 100% | 32% | 0.5 | |  |
| S10 | F | 41 | 1185 | R | ADVANCED | 4.5 | 8% | 2% | 0.4 | |  |
|  |  |  |  | L | ADVANCED | 4.0 | 6% | 2% | 0.3 | |  |
| S11 | F | 38 | 1531 | R | ADVANCED | 4.0 | 21% | 3% | 0.3 | |  |
|  |  |  |  | L | MODERATE | 5.0 | 25% | 4% | 0.3 | |  |
| S12 | F | 48 | 1350 | R | MILD | 10.0 | 50% | 26% | 0.3 | |  |
|  |  |  |  | L | ADVANCED | 4.0 | 88% | 13% | 0.4 | |  |
| S13 | F | 43 | 766 | R | MODERATE | 5.0 | 0% | 0% | 0.7 | |  |
|  |  |  |  | L | MODERATE | 6.0 | 0% | 0% | 0.4 | |  |
| S14 | M | 28 | 882 | R | MODERATE | 6.0 | 96% | 4% | 0.4 | |  |
|  |  |  |  | L | MODERATE | 5.0 | 100% | 2% | 0.5 | |  |
| S15 | M | 29 | 2841 | R | MODERATE | 7.0 | 96% | 45% | 0.2 | |  |
|  |  |  |  | L | MODERATE | 5.0 | 92% | 29% | 0.1 | |  |
| S16 | F | 52 | 2277 | R | MODERATE | 7.0 | 4% | 0% | 0.15 | |  |
|  |  |  |  | L | MODERATE | 6.0 | 0% | 0% | 0.05 | |  |
| S17 | F | 22 | 1875 | R | MODERATE | 7.0 | 13% | 12% | 0.3 | |  |
|  |  |  |  | L | MODERATE | 7.0 | 17% | 12% | 0.2 | |  |
| S18 | M | 46 | 1264 | R | MODERATE | 7.0 | 96% | 13% | 0.1 | |  |
|  |  |  |  | L | MILD | 10.0 | 100% | 15% | 0.5 | |  |
| S19 | F | 64 | 1688 | R | MILD | 12.0 | 96% | 33% | -0.2 | |  |
|  |  |  |  | L | MODERATE | 8.0 | 96% | 45% | -0.2 | |  |
| S20 | M | 42 | 1723 | R | MODERATE | 9.0 | 75% | 2% | 0.2 | |  |
|  |  |  |  | L | MILD | 10.0 | 92% | 6% | 0.1 | |  |
| S21 | M | 38 | 2199 | R | MILD | 12.0 | 33% | 47% | 0.1 | |  |
|  |  |  |  | L | MILD | 12.0 | 33% | 46% | 0.1 | |  |
| S22 | M | 51 | 1803 | R | MILD | 12.0 | 100% | 9% | 0.2 | |  |
|  |  |  |  | L | MILD | 14.0 | 96% | 34% | 0.15 | |  |
| S23 | F | 23 | 1811 | R | MILD | 15.0 | 100% | 4% | 0.2 | |  |
|  |  |  |  | L | MILD | 12.0 | 100% | 5% | 0.4 | |  |
| S24 | F | 57 | 1826 | R | MILD | 15.0 | 100% | 13% | 0 | |  |
|  |  |  |  | L | MILD | 20.0 | 100% | 10% | 0 | |  |
| S25 | M | 25 | 1866 | R | MILD | 15.0 | 100% | 1% | 0.3 | |  |
|  |  |  |  | L | MILD | 20.0 | 100% | 8% | 0.4 | |  |
| S26 | F | 51 | 1481 | R | MILD | 15.0 | 100% | 13% | 0.05 | |  |
|  |  |  |  | L | MILD | 20.0 | 100% | 19% | 0.05 | |  |
| S27 | M | 24 | 1964 | R | MILD | 20.0 | 100% | 79% | -0.1 | |  |
|  |  |  |  | L | MILD | 20.0 | 100% | 77% | -0.1 | |  |
| S28 | F | 34 | 1847 | R | HEALTHY CONTROL | 100.0 | 100% | 100% | -0.2 | |  |
|  |  |  |  | L | HEALTHY CONTROL | 100 | 100% | 100% | -0.2 | |  |
| S29 | F | 22 | 2026 | R | HEALTHY CONTROL | 100 | 100% | 100% | -0.2 | |  |
|  |  |  |  | L | HEALTHY CONTROL | 100 | 100% | 100% | -0.2 | |  |
| S30 | F | 45 | 2139 | R | HEALTHY CONTROL | 100 | 100% | 100% | -0.2 | |  |
|  |  |  |  | L | HEALTHY CONTROL | 100 | 100% | 100% | -0.2 | |  |
| S31 | F | 35 | 2148 | R | HEALTHY CONTROL | 100 | 100% | 100% | -0.2 | |  |
|  |  |  |  | L | HEALTHY CONTROL | 100 | 100% | 100% | -0.2 | |  |
| S32 | M | 58 | 1667 | R | HEALTHY CONTROL | 100 | 100% | 100% | -0.2 | |  |
|  |  |  |  | L | HEALTHY CONTROL | 100 | 100% | 100% | -0.2 | |  |
| S33 | M | 39 | 1658 | R | HEALTHY CONTROL | 100 | 100% | 100% | -0.2 | |  |
|  |  |  |  | L | HEALTHY CONTROL | 100 | 100% | 100% | -0.2 | |  |
| S34 | M | 62 | 2434 | R | HEALTHY CONTROL | 100 | 100% | 100% | -0.1 | |  |
|  |  |  |  | L | HEALTHY CONTROL | 100 | 100% | 100% | -0.1 | |  |
| S35 | M | 61 | 2233 | R | HEALTHY CONTROL | 100 | 100% | 100% | 0 | |  |
|  |  |  |  | L | HEALTHY CONTROL | 100% | 100% | 100% | 0 | |  |

M, male; F, female; R, right; L, left; logMAR, logarithm of the minimum angle of resolution.

The gray color-coded columns are the dominant eyes.
