# Supplementary material for: Importance of two-dimensional gaze analyses in the assessment of reading performance in patients with retinitis pigmentosa
Source: PLoS One. 2022 Dec 14;17(12):e0278682. doi: 10.1371/journal.pone.0278682 (PMC9750004; doi:10.1371/journal.pone.0278682)
Supplement: S2 Table — (DOCX) [file pone.0278682.s004.docx]

**S2 Table. Analysis of ocular motility measured with an eye mark recorder for all participants**

| Subject No. | Gender | Age (years) | Analysis of ocular motility | | | | | | | | | | | |
| --- | --- | --- | --- | --- | --- | --- | --- | --- | --- | --- | --- | --- | --- | --- |
|  |  |  | Fixation(sec) | | | Saccades(%) | | | Length of eye movements | | | Number of Errors | | Task  performance  time  (sec) |
|  |  |  | Total duration | Mean duration | Fixation counts | Parallel (Progress) | Regressive SC | 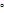Rotation SC | ΣTotal (degrees) | ΣX (degrees) | ΣY (degrees) | X | Y |  |
| S1 | F | 43 | 12.33 | 1.37 | 9 | 69.2 | 15.4 | 15.4 | 153.5 | 107.3 | 86.1 | 25 | 24 | 13.247 |
|  |  |  | 12.58 | 0.8387 | 15 | 21.1 | 10.5 | 68.4 | 251.4 | 182.4 | 225.2 | 45 | 28 |  |
| S2 | M | 38 | 10.034 | 0.5017 | 20 | 4.2 | 4.2 | 91.6 | 321.3 | 287.4 | 252.5 | 500 | 568 | 18.886 |
|  |  |  | 10.0468 | 0.3464 | 29 | 7.3 | 2.4 | 90.3 | 483.6 | 353.3 | 317.1 | 278 | 256 |  |
| S3 | M | 41 | 10.98 | 0.9982 | 11 | 68.8 | 25 | 8.3 | 198.3 | 155.8 | 78.5 | 4 | 4 | 12.579 |
|  |  |  | 12.279 | 1.0232 | 12 | 68.8 | 25 | 8.3 | 225.0 | 150.8 | 115.1 | 5 | 0 |  |
| S4 | F | 37 | 6.478 | 0.2699 | 24 | 7.7 | 15.4 | 76.9 | 278.2 | 292.5 | 260.8 | 244 | 272 | 8.475 |
|  |  |  | 3.617 | 0.4521 | 8 | 11.1 | 33.3 | 55.6 | 362.8 | 372.0 | 234.3 | 162 | 106 |  |
| S5 | M | 63 | 6.174 | 0.441 | 14 | 62.5 | 18.8 | 18.7 | 193.8 | 144.8 | 88.9 | 0 | 0 |  |
|  |  |  | 6.174 | 0.441 | 14 | 68.8 | 18.8 | 12.4 | 216.3 | 166.3 | 91.3 | 3 | 3 | 9.076 |
| S6 | F | 55 | 6.941 | 0.5784 | 12 | 53.3 | 13.3 | 33.4 | 286.5 | 200.0 | 165.0 | 6 | 6 |  |
|  |  |  | 7.258 | 0.5583 | 13 | 62.5 | 18.8 | 18.7 | 168.1 | 111.7 | 117.6 | 21 | 18 | 7.841 |
| S7 | F | 63 | 6.405 | 0.3203 | 20 | 47.1 | 41.2 | 11.7 | 533.5 | 517.0 | 81.6 | 0 | 0 | 6.64 |
|  |  |  | 7.474 | 0.5338 | 14 | 17.9 | 10.7 | 71.4 | 437.7 | 392.2 | 153.7 | 220 | 1 |  |
| S8 | M | 64 | 5.039 | 0.4889 | 9 | 66.7 | 33.3 | 0 | 97.3 | 77.2 | 35.3 | 0 | 0 |  |
|  |  |  | 4.706 | 0.4706 | 10 | 33.3 | 16.7 | 50 | 108.0 | 61.7 | 62.3 | 5 | 5 | 6.273 |
| S9 | F | 41 | 4.656 | 0.4233 | 11 | 71.4 | 14.3 | 14.3 | 120.0 | 81.5 | 57.5 | 13 | 13 | 6.073 |
|  |  |  | 5.322 | 0.4838 | 11 | 78.6 | 14.3 | 7.1 | 166.5 | 126.7 | 97.1 | 22 | 21 |  |
| S10 | F | 41 | 8.286 | 0.5178 | 16 | 66.7 | 16.7 | 16.6 | 135.0 | 97.2 | 61.8 | 0 | 0 | 8.308 |
|  |  |  | 8.177 | 0.6814 | 11 | 76.5 | 17.6 | 5.9 | 159.9 | 89.7 | 125.1 | 3 | 1 |  |
| S11 | F | 38 | 7.074 | 0.7074 | 10 | 45.5 | 18.2 | 36.3 | 125.7 | 75.8 | 77.5 | 11 | 13 |  |
|  |  |  | 6.39 | 0.71 | 9 | 63.6 | 18.2 | 18.2 | 113.7 | 83.5 | 47.9 | 0 | 0 | 7.741 |
| S12 | F | 48 | 7.324 | 0.8138 | 9 | 61.5 | 15.4 | 33.1 | 129.6 | 97.4 | 115.4 | 49 | 47 | 7.641 |
|  |  |  | 7.574 | 0.8416 | 9 | 53.8 | 15.4 | 20.8 | 214.1 | 208.0 | 168.6 | 32 | 34 |  |
| S13 | F | 43 | 6.824 | 0.6824 | 10 | 90 | 10 | 0 | 166.9 | 132.5 | 67.4 | 3 | 3 |  |
|  |  |  | 6.925 | 0.6925 | 10 | 90 | 10 | 0 | 147.7 | 100.6 | 68.4 | 7 | 7 | 7.641 |
| S14 | M | 28 | 5.107 | 0.3928 | 13 | 72.2 | 27.8 | 0 | 149.6 | 169.8 | 80.5 | 16 | 13 | 6.607 |
|  |  |  | 6.023 | 0.5019 | 12 | 76.5 | 23.5 | 0 | 160.3 | 117.3 | 73.0 | 12 | 12 |  |
| S15 | M | 29 | 3.136 | 0.392 | 8 | 50 | 50 | 0 | 125.0 | 95.9 | 61.9 | 12 | 7 | 5.772 |
|  |  |  | 4.772 | 0.3077 | 12 | 71.4 | 21.4 | 7.2 | 163.1 | 138.0 | 54.7 | 6 | 8 |  |
| S16 | F | 52 | 5.072 | 0.4611 | 11 | 81.8 | 18.2 | 0 | 88.0 | 69.8 | 26.8 | 0 | 0 | 5.439 |
|  |  |  | 5.405 | 0.4158 | 13 | 76.9 | 15.4 | 7.7 | 105.8 | 71.3 | 47.1 | 0 | 0 |  |
| S17 | F | 22 | 4.788 | 0.399 | 12 | 37.5 | 12.5 | 50 | 167.1 | 173.1 | 97.4 | 7 | 10 |  |
|  |  |  | 4.771 | 0.4337 | 11 | 37.5 | 25 | 37.5 | 117.3 | 142.7 | 73.4 | 13 | 15 | 4.638 |
| S18 | M | 46 | 5.7825 | 0.4136 | 14 | 76.5 | 23.5 | 0 | 114.9 | 102.4 | 103.6 | 32 | 3 | 6.64 |
|  |  |  | 2.401 | 0.2668 | 9 | 38.5 | 15.4 | 46.1 | 272.1 | 193.6 | 249.9 | 133 | 112 |  |
| S19 | F | 64 | 6.407 | 0.6407 | 10 | 50 | 7.1 | 42.9 | 119.9 | 83.2 | 62.7 | 41 | 41 |  |
|  |  |  | 6.707 | 0.7452 | 9 | 9.1 | 0 | 90.9 | 99.1 | 78.3 | 57.8 | 41 | 42 | 6.807 |
| S20 | M | 42 | 5.457 | 0.496 | 11 | 64.3 | 14.3 | 21.4 | 151.6 | 116.7 | 57.4 | 16 | 16 |  |
|  |  |  | 5.34 | 0.534 | 10 | 42.9 | 14.3 | 42.8 | 154.3 | 119.6 | 55.7 | 51 | 52 | 6.54 |
| S21 | M | 38 | 3.019 | 0.3774 | 8 | 80 | 10 | 10 | 232.6 | 257.5 | 103.0 | 44 | 43 | 6.44 |
|  |  |  | 4.338 | 0.482 | 9 | 70 | 10 | 20 | 298.7 | 223.3 | 150.7 | 38 | 38 |  |
| S22 | M | 51 | 6.172 | 0.6172 | 10 | 70 | 30 | 0 | 93.6 | 72.1 | 35.2 | 2 | 2 |  |
|  |  |  | 6.74 | 0.674 | 10 | 70 | 30 | 0 | 132.6 | 100.3 | 57.8 | 3 | 3 | 5.172 |
| S23 | F | 23 | 4.588 | 0.4588 | 10 | 90.9 | 9.1 | 0 | 87.8 | 71.7 | 47.6 | 6 | 5 | 5.405 |
|  |  |  | 5.772 | 0.5772 | 10 | 76.9 | 23.1 | 0 | 83.5 | 70.8 | 21.8 | 146 | 146 |  |
| S24 | F | 57 | 5.673 | 0.5157 | 11 | 57.9 | 26.3 | 15.8 | 136.0 | 116.9 | 36.9 | 0 | 0 | 5.639 |
|  |  |  | 4.923 | 0.3787 | 13 | 39.1 | 17.4 | 43.5 | 142.7 | 117.6 | 44.4 | 0 | 0 |  |
| S25 | M | 25 | 5.07 | 0.507 | 10 | 81.8 | 18.2 | 0 | 105.4 | 75.1 | 46.8 | 0 | 0 | 5.439 |
|  |  |  | 5.054 | 0.5054 | 10 | 81.8 | 18.2 | 0 | 115.6 | 81.4 | 54.1 | 0 | 0 |  |
| S26 | F | 51 | 6.874 | 0.7637 | 9 | 66.7 | 33.3 | 0 | 187.4 | 183.7 | 129.4 | 5 | 5 |  |
|  |  |  | 6.758 | 0.7509 | 9 | 72.7 | 27.3 | 0 | 139.9 | 97.4 | 32.9 | 11 | 9 | 7.274 |
| S27 | M | 24 | 5.506 | 0.50055 | 11 | 83.3 | 16.7 | 0 | 88.8 | 66.2 | 31.6 | 0 | 0 |  |
|  |  |  | 5.873 | 0.5873 | 10 | 81.8 | 18.2 | 0 | 88.2 | 66.2 | 32.5 | 0 | 0 | 5.506 |
| S28 | F | 34 | 6.508 | 0.5916 | 11 | 66.7 | 16.7 | 16.6 | 107.3 | 86.4 | 37.3 | 9 | 9 | 7.207 |
|  |  |  | 6.458 | 0.6458 | 10 | 58.3 | 16.7 | 25 | 148.9 | 120.4 | 61.9 | 11 | 11 |  |
| S29 | F | 22 | 5.823 | 0.5294 | 11 | 64.3 | 21.4 | 14.3 | 168.7 | 119.9 | 151.2 | 18 | 2 |  |
|  |  |  | 5.957 | 0.5415 | 11 | 61.5 | 23.1 | 15.4 | 166.2 | 86.6 | 103.9 | 15 | 15 | 6.573 |
| S30 | F | 45 | 5.306 | 0.4422 | 12 | 76.9 | 23.1 | 0 | 109.6 | 85.8 | 42.6 | 0 | 0 | 6.073 |
|  |  |  | 5.272 | 0.4793 | 11 | 78.6 | 21.4 | 0 | 113.2 | 85.5 | 45.6 | 0 | 0 |  |
| S31 | F | 35 | 4.138 | 0.4598 | 9 | 88.9 | 11.1 | 0 | 99.3 | 83.2 | 40.4 | 38 | 5 | 5.973 |
|  |  |  | 3.937 | 0.3937 | 10 | 90.9 | 9.1 | 0 | 152.7 | 123.2 | 106.4 | 10 | 8 |  |
| S32 | M | 58 | 5.472 | 0.5472 | 10 | 90 | 10 | 0 | 110.1 | 88.7 | 33.9 | 0 | 0 | 8.041 |
|  |  |  | 5.456 | 0.5456 | 11 | 92.3 | 7.7 | 0 | 116.4 | 90.5 | 39.4 | 0 | 0 |  |
| S33 | M | 39 | 6.157 | 0.7696 | 8 | 80 | 20 | 0 | 82.0 | 67.3 | 23.1 | 0 | 0 | 6.673 |
|  |  |  | 6.316 | 0.9023 | 7 | 70 | 30 | 0 | 87.5 | 67.9 | 30.1 | 0 | 0 |  |
| S34 | M | 62 | 4.788 | 0.532 | 9 | 88.9 | 11.1 | 0 | 85.2 | 71.2 | 34.7 | 6 | 5 |  |
|  |  |  | 5.072 | 0.634 | 8 | 88.9 | 11.1 | 0 | 78.3 | 58.3 | 40.1 | 5 | 3 | 6.106 |
| S35 | M | 61 | 6.508 | 0.5916 | 11 | 76.5 | 23.5 | 0 | 126.2 | 95.0 | 66.5 | 6 | 3 | 6.94 |
|  |  |  | 6.458 | 0.6458 | 10 | 75 | 25 | 0 | 139.7 | 106.6 | 60.4 | 8 | 8 |  |

F, female; M, male; SC, saccade; The gray color-coded column are the dominant eyes. ΣX (degree); total eye movement distance in horizontal direction; ΣY (degree); total eye movement distance in vertical direction.
